# Age-Related Variations in the Clinical Presentation and Treatment Outcomes of New-Onset GPA: A Longitudinal Study

**DOI:** 10.3390/jcm14051544

**Published:** 2025-02-25

**Authors:** Malgorzata Potentas-Policewicz, Dariusz Gawryluk, Elzbieta Wiatr, Justyna Fijolek

**Affiliations:** 1 Department of Geriatrics, Dr Anna Gostynska Wolski Hospital, 01-211 Warsaw, Poland; potentasm@wp.pl; 2 Third Department of Pneumonology and Oncology, National Tuberculosis and Lung Diseases Research Institute, 01-138 Warsaw, Poland; dpgawryluk@gmail.com (D.G.); e.wiatr@igichp.edu.pl (E.W.)

**Keywords:** granulomatosis with polyangiitis, age-related clinical variations, corticosteroid therapy, lung involvement in vasculitis, cyclophosphamide dosing, peripheral neuropathy

## Abstract

**Background/Objectives**: This study compares the clinical features and treatment outcomes of granulomatosis with polyangiitis (GPA) based on age at onset. **Methods**: A retrospective longitudinal cohort of patients with GPA diagnosed between January 1978 and December 2015 was analyzed, stratified by age at diagnosis: ≤30 years (young group), 31–59 years (middle-aged group), and ≥60 years (older group). The comparative analysis included demographic data, organ involvement, laboratory results, anti-neutrophil cytoplasmic antibody (ANCA) status, comorbidities, treatments, and outcomes. **Results**: The analysis included 264 patients newly diagnosed with GPA. Older patients exhibited significantly higher rates of peripheral neuropathy and liver involvement. They had more severe lung diseases and required lung biopsies more frequently. Patients in the middle-aged group exhibited the highest likelihood of severe anemia. Peripheral neuropathy was more common in this group than in younger patients, and their lung disease was less severe than in older patients but more severe than in younger patients. Young patients exhibited mild disease with the least severe lung involvement, mild anemia, and highest albumin levels. Baseline comorbidities and post-treatment adverse events increased significantly with age at diagnosis. Treatment strategies and efficacy were similar across groups, although older patients tended to receive lower initial doses of cyclophosphamide and corticosteroids. **Conclusions**: Age at diagnosis influenced GPA clinical characteristics. While the treatment did not vary significantly by age at onset, tailoring therapy to a patient’s age is crucial to optimize outcomes and minimize complications.

## 1. Introduction

Granulomatosis with polyangiitis (GPA) is a rare systemic disease characterized by necrotizing vasculitis of small-to-medium-sized vessels and granulomatous inflammation, primarily affecting the upper and lower respiratory tracts [1]. It is classified as an anti-neutrophil cytoplasmic antibody (ANCA)–associated vasculitis (AAV) [2]. GPA is typically associated with proteinase 3 (PR3) epitopes, while other entities like microscopic polyangiitis (MPA) and eosinophilic granulomatosis with polyangiitis (EGPA) are linked to myeloperoxidase (MPO)-ANCA or negative ANCA [3]. Although AAV is a separate syndrome, significant phenotypic overlap exists, particularly between GPA and MPA [4]. The target antigen of ANCA is not always discriminatory; approximately 9% of patients with GPA are MPO-ANCA positive [5], whereas up to 40% of patients with MPA may be PR3-ANCA positive [6]. This overlap in clinical presentations, combined with distinct genetic associations, [7], has prompted discussions on reclassifying AAV based on ANCA type (PR3 versus MPO) rather than clinical phenotypes (GPA versus MPA).

Although AAV is rare, its incidence has increased over the past 40 years, with GPA being the most common form [8]. A recent French study reported an age-standardized incidence of 0.5/100,000 person-years for GPA and a prevalence of 10/100,000 person-years compared to 0.3/100,000 and 4/100,000 for MPA, respectively [9]. However, recent data from Sweden indicate a stable AAV incidence over 23 years (annual incidence: 15.4/million for GPA, compared to 12.8 for MPA and 1.8 for EGPA) and an increasing prevalence, likely due to better management and treatment outcomes for AAV, which have improved survival rates [10]. In addition to the increasing incidence, the peak age at diagnosis has shifted, with the highest incidence of AAV observed in individuals aged 70–84 years in Sweden (96/million) [10]. Another UK study reported peak incidence in patients over 80 years [11]. However, GPA and MPA display age discrepancies at diagnosis, with GPA peaking at 45–55 years and MPA at 65–70 years [12].

GPA generally affects the upper respiratory tract, lungs, and kidneys, but any organ can be involved [13]. The disease spectrum and severity are heterogeneous, ranging from indolent, localized disease to fulminant, multiorgan vasculitis, leading to death. The course of the disease in individual patients remains unclear. Limited reports suggest that the clinical characteristics of GPA may vary across the lifespan, but the findings are inconsistent [14,15]. Additionally, despite potential differences, most studies compared older and younger patients without distinguishing between young and middle-aged groups. Data on the age-related clinical expression of GPA, especially in cohorts diagnosed and treated in pneumological departments, remain scarce [16]. This study aimed to compare clinical manifestations and treatment outcomes in a large monocentric cohort of patients with GPA stratified by age of onset (≤30 years, 31–59 years, ≥60 years). The findings from this study may provide insights into early diagnosis and optimized treatment strategies to reduce organ damage and improve the outcomes of GPA.

## 2. Materials and Methods

### 2.1. Ethics

This retrospective study was approved by the Bioethics Committee of the National Tuberculosis and Lung Disease Research Institute (NTbLDRI), No. KB-5/2021, on April 13, 2021. All procedures followed in this study adhered to the ethical standards of the institutional and national research committees, as well as the principles outlined in the 1964 Declaration of Helsinki and its subsequent amendments.

Due to the retrospective nature of this study, the Bioethics Committee at NTbLDRI waived the requirement for written informed consent from the study participants.

### 2.2. Patients and Analyzed Data

This study included patients diagnosed with new-onset GPA diagnosed between January 1978 and December 2015 at the pulmonary department of NTbLDRI, who met the American College of Rheumatology (ACR) 1990 classification criteria [17] and/or the revised Chapel Hill nomenclature [1]. Patients were divided into three age groups: ≤30 years, 31–59 years, and ≥60 years. The following baseline data were used for analysis and comparison among the groups: demographic data, detailed organ involvement, laboratory test results (including full blood count, total protein and albumin concentrations, acute phase reactants, creatinine levels, 24-h proteinuria, hematuria, liver enzymes, blood gas analysis, ANCA test results, and nasal carriage of *Staphylococcus aureus* (*S. aureus*)), presence of comorbidities, treatment data (agents used to induce and maintain disease remission, systemic corticosteroid (CS) use and dose, duration of immunosuppression (IS), and other treatment procedures), and treatment outcomes (response, duration of remission, relapses, adverse events (AEs)), as well as early damage (assessed using the vasculitis damage index (VDI) score after the completion of the first treatment cycle). The observation period lasted until the end of 2020, ensuring a minimum duration of 5 years.

### 2.3. Organ Involvement Assessment

Organ involvement was assessed through clinical signs, laboratory findings, imaging results, and histological examination. Gastrointestinal involvement also included liver involvement, defined by baseline liver enzymes levels ≥3 times the upper limit of normal, after excluding other causes and in cases where enzyme levels decreased or normalized during GPA treatment. Renal involvement was recognized by abnormal urinalysis (hematuria > 5 red blood cells/mm), proteinuria (>0.5 g/day), or an increase in serum creatinine above the normal range (adjusted for age and sex) or worsening by >30%.

### 2.4. Disease Activity Assessment

Disease activity at diagnosis was assessed using the Birmingham Vasculitis Activity Score (BVAS) version specific to Wegener’s granulomatosis (BVAS/WG) [18]. The BVAS scores were retrospectively calculated for patients diagnosed before 2000.

### 2.5. ANCA Assessment

ANCA positivity was determined using indirect immunofluorescence (IF) and/or enzyme-linked immunosorbent assay (ELISA) to identify MPO or PR3 antigens in serum at diagnosis. A positive ANCA result was defined as ≥20 μ/mL.

### 2.6. Definitions

Localized disease was defined as disease confined to the ENT and/or lower respiratory tract (mainly subglottic stenosis (SGS)). Remission, relapse, and treatment failure (including refractory disease or causes requiring discontinuation of immunosuppression (IS), such as severe infection, damage, or side effects) were defined according to EULAR recommendations [19,20]. Minimally persistent AAV activity was defined as a condition in which a patient, despite otherwise well-controlled vasculitis, exhibited minor persistent symptoms such as arthralgia, fatigue, or atrophic rhinitis. These symptoms did not necessitate an escalation of treatment, except for a possible slight increase in corticosteroid (CS) therapy [21]. A delay in diagnosis was defined as the time from the first symptoms to the final diagnosis of GPA. Carriage of *S. aureus* was defined as a positive result from three consecutive nasal swabs [22]. Comorbidities included diseases and conditions that occurred at least two years before the diagnosis of GPA. Early damage was calculated using the Vasculitis Damage Index (VDI) score and assessed after completion of the first treatment cycle.

### 2.7. Statistical Analysis

The distribution of continuous variables was assessed using the Shapiro–Wilk test. As most continuous variables were not normally distributed, they were presented as medians with interquartile ranges (IQRs). Categorical variables were presented as counts and percentages.

Comparisons among the three patient groups were performed using the Kruskal–Wallis test for continuous variables and the chi-square test for categorical variables. When differences were statistically significant (*p* < 0.05) or approached significance (*p* close to 0.05), the Bonferroni correction was applied to determine which age groups showed significant differences. According to this correction, *p* < 0.017 was considered statistically significant, while *p*-values between 0.017 and 0.05 were interpreted as approaching significance.

All statistical analyses were conducted using STATISTICA PL software (version 13; Tulsa, OK, USA).

## 3. Results

### 3.1. General Characteristics of Study Patients

This study included 264 patients with GPA: 59 diagnosed at age ≤30 years (young group), 156 diagnosed from 31 to 59 years (middle-aged group), and 49 diagnosed at age ≥60 years (older group). The median follow-up time was 12.5 years (IQR 8.6–17.8), which differed significantly among the age groups (*p* = 0.001), being significantly shorter in the older group compared with the younger group (median, 10.0 vs. 14.7 years; *p* = 0.001). Among all the patients, 52% were female, with no significant differences between the groups. However, the percentage of women was highest in the older group (61% vs. 56% in the younger group and 47% in the middle-aged group). The median BVAS/WG score was 8 points (IQR 5–12) and tended to be significantly higher in the middle-aged group (*p* = 0.029).

The lungs were the most frequently affected organs (92%), followed by the upper respiratory tract (84%), kidneys (57%), lower respiratory tract (43%), skin (29%), eyes (24%), joints (23%), nervous system (16%), digestive tract (11%), and heart (6%). The majority of patients (93%; 246/264) had multiorgan manifestations, with 56% (147/264) of cases involving more than three organs, with no significant differences between the age groups (*p* = 0.211). Only 6% (17/264) of patients had localized GPA, with a comparable distribution in the individual age groups (*p* = 0.452).

Histological confirmation of GPA (at least one feature attributable to GPA) was obtained in 70% of patients (184/264). The most commonly biopsied organs included the nose (30%), lungs (22%), bronchi (18%), trachea (7%), skin (6%), kidneys (3%), oral cavity (2%), and digestive tract (1%). The distribution of biopsy procedures was generally similar, except for lung biopsies, performed significantly more frequently in older patients than in younger patients (31% vs. 10%, *p* = 0.008). The median time to diagnosis delay for all patients was 4 months (IQR 2–10), with a strong tendency to delays in the older group compared to the younger and middle-aged groups (4 vs. 6 and 6 months; *p* = 0.025 and *p* = 0.048, respectively).

### 3.2. Comparison of Individual Age Onset Groups

#### Clinical Manifestations

A comparative analysis revealed differences in peripheral neuropathy, liver involvement, hearing loss (related to vasculitis), and arthritis. Patients in the middle-aged and older groups experienced significantly more peripheral neuropathy than those in the younger age group (10% vs. 0%, *p* = 0.014 and 10% vs. 0%, *p* = 0.012, respectively). In addition, older patients had significantly more frequent liver involvement than middle-aged patients (12% vs. 3%, *p* = 0.014). Although the frequency of ear, nose, and throat (ENT) symptoms was comparable among all groups (*p* = 0.477), hearing loss at baseline related to vasculitis occurred more frequently in older patients than in younger patients, with this difference approaching statistical significance (14% vs. 2%, *p* = 0.034). Conversely, young and middle-aged patients experienced more arthritis symptoms than older patients (25% and 27% vs. 10%, *p* = 0.043 and *p* = 0.017, respectively). Additionally, the middle-aged group tended to have higher BVAS/WG scores than the older group (*p* = 0.029), but not the young group. Detailed clinical characteristics of study patients with pairwise comparison across age groups are shown in Table 1.

### 3.3. Laboratory Tests

**Figure 1:**
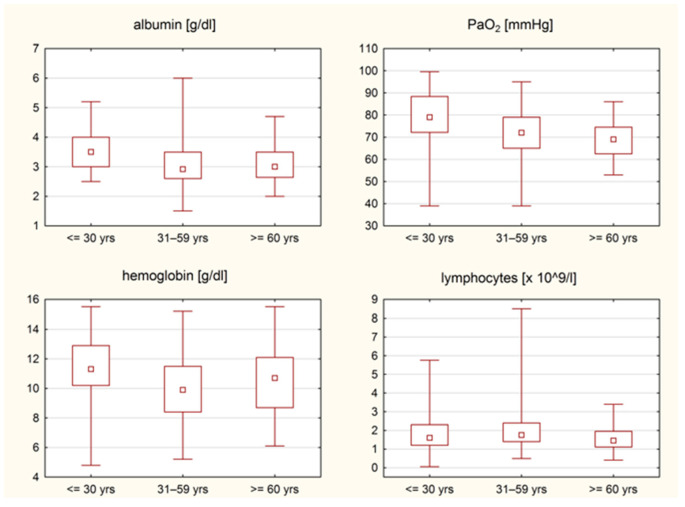
Significant differences in laboratory tests results across age groups.

Differences in laboratory findings are summarized in Table 2. Young patients had significantly higher hemoglobin levels compared to the middle-aged group (median, 11.3 g/dL vs. 9.9 g/dL; *p* = 0.003), and significantly higher albumin concentrations compared to both the middle-aged (median, 3.5 g/dL vs. 2.9 g/dL; *p* = 0.001) and older groups (median, 3.5 g/dL vs. 3.0 g/dL; *p* = 0.016). In contrast, the lowest lymphocyte blood count at baseline was observed in older patients, with significant differences compared to the middle-aged group but not the young group (median, 1.5 × 10^9^/L vs. 1.8 × 10^9^/L; *p* = 0.013). Finally, patients diagnosed as older or middle-aged had significantly lower partial pressure of oxygen (PaO_2_) at baseline compared to the young group (median, 69.0 vs. 79.0 mmHg, and 72.0 vs. 79.0 mmHg, respectively; *p* = 0.001 and *p* < 0.001, respectively) (Figure 1).

The median creatinine level at baseline in all patients was 0.9 mg/dL (IQR 0.8–1.3) and increased with age of GPA onset (median, 0.8 mg/dL, 0.9 mg/dL, and 1.0 mg/dL). However, although the initial analysis suggested significant differences between the groups (*p* = 0.029), a detailed comparison showed a strong tendency for lower creatinine levels and better renal function in the young group than in the middle-aged group (*p* = 0.039) but not in the older group (*p* = 0.083). The highest CRP concentrations were observed in the middle-aged patients and tended to be significantly higher compared to the young group (median, 79.6 mg/mL vs. 18.6 mg/mL; *p* = 0.046).

### 3.4. ANCA Status

ANCA data were available for 262 patients. Of these, 88% (232/262) had positive ANCA test results, whereas 11.5% (30/262) were ANCA-negative. cANCA was detected in 71% (185/262) of the patients, while pANCA was detected in 6% (16/262). There were no significant differences in the frequencies of ANCA or cANCA. However, pANCA positivity was significantly more common in the older group than in the young group (14% vs. 2%, *p* = 0.013) and tended to be more common than in the middle-aged group (14% vs. 5%, *p* = 0.035).

Data on the ANCA target antigens (PR3 or MPO) were available for 96 patients. PR3-ANCA was detected in 88% (84/96) of patients and MPO-ANCA in 5% (5/96). There were no significant differences between age groups in the distribution of PR3- and MPO-ANCA (*p* = 0.548 and *p* = 0.280, respectively).

### 3.5. Carriage of S. Aureus

Data on nasal carriage of *S. aureus* were available for 234 patients. *S. aureus* was present in 43% of cases (100/234) and was more frequently found in the young and middle-aged groups than in the older group (47% and 46% vs. 28%, respectively). These differences were close to being significant (*p* = 0.051 and *p* = 0.037, respectively).

### 3.6. Comorbidities

Fifty-eight percent of patients (153/264) had comorbidities at the time of GPA diagnosis (Table 3). The number of comorbidities increased significantly with age at GPA onset (36% in the young, 58% in the middle-aged, and 88% in the older group; *p* = 0.003, *p* < 0.001, and *p* < 0.001, respectively). Among these, hypertension (HTN), diabetes mellitus (DM), and cancer were significantly more common in the older group than in the other age groups (*p* < 0.001, *p* = 0.003, and *p* = 0.014, respectively). Older patients were also more likely to present with depression (10% vs. 2% in middle-aged and 0% in young adults). However, these differences were not statistically significant, and only a strong tendency was observed (*p* = 0.040 and *p* = 0.029, respectively).

Twenty-seven percent of patients (70/264) had various other comorbidities such as thyroid diseases, gastritis, gastroesophageal reflux disease (GERD), inflammatory bowel disease (IBD), uterine fibroids, benign prostatic hyperplasia (BPH), or cholecystolithiasis. These comorbidities were most frequent in the middle-aged group (32% vs. 17% in young and 20% in older patients, respectively), with a tendency to be significantly differed compared with the young group (*p* = 0.028).

### 3.7. Treatment and Outcomes

Treatment strategies and efficacy across age groups are shown in Table 4. Eighty-six percent of patients (228/264) received standard immunosuppressive (IS) treatment based on systemic corticosteroids (CS) and cyclophosphamide (CYC), ten percent (25/264) received only systemic CS, and four percent (10/264) received other IS drugs (five received etoposide, two received cyclosporine, and three received mycophenolate mofetil). The median starting dose of oral CS was 1 mg/kg/day of prednisolone equivalent, but older patients received a lower dose than the young group (median, 0.8 mg vs. 1.0 mg, *p* = 0.027). The median total duration of CS use was 19 months (IQR 12–24), and it was comparable across age groups (*p* = 0.855).

Most patients (80%, 210/264) received oral CYC at a median dose of 2.0 mg/kg/day. There was a stronger tendency toward lower CYC doses in older patients than in younger patients (*p* = 0.047). The median duration of CYC treatment was 8.8 months (IQR 5.5–15.8), and the median cumulative dose was 28 g (IQR 15–47), with both parameters comparable across groups (*p* = 0.579 and *p* = 0.826, respectively). However, older patients were treated for a shorter time compared to young (median, 8.0 vs. 9.5 months) and received the lowest cumulative dose of CYC (median, 26 vs. 31 g in the young group and 27 g in the middle-aged group).

The most common drug used for maintenance treatment was CYC (40%; 105/264), followed by azathioprine (36%; 96/264) and methotrexate (12%; 31/264), while 12% of patients (32/264) continued only CS. There were no significant differences in remission maintenance drug distribution between the age groups (*p* = 0.518). The overall median total IS duration was 17 months [11–24 months], which was also comparable between age groups (*p* = 0.463).

During the observation period, no patient received rituximab as a first-line treatment for newly diagnosed GPA. However, 7% of the patients (18/264) received rituximab for relapsing and/or refractory disease, with no significant differences between the groups (*p* = 0.682).

Regarding other therapeutic procedures (listed in Table 4), significant differences were found only in the administration of trimethoprim/sulfamethoxazole (T/S), which was significantly more common in the younger group than in the middle-aged and older groups (37% vs. 17% and 16%, respectively; *p* = 0.002 and *p* = 0.015). Additionally, blood transfusions were more frequent in the middle-aged group than in the older group, with a strong tendency toward significance (15% vs. 4%; *p* = 0.038).

Disease remission was achieved in 78% of patients (206/264). There were no significant differences in treatment efficacy or ANCA seroconversion across age groups (*p* = 0.564 and *p* = 0.533, respectively).

During the follow-up period, 58% of patients (153/264) experienced a relapse, with no statistically significant differences observed between the age groups (*p* = 0.520). The median number of relapses was 1 (IQR 0–2), with 22% of patients (59/264) experiencing three or more relapses. The median time from treatment completion to the first relapse was 11 months (IQR 4–26), with no significant differences between the groups (*p* = 0.675). However, relapse-free survival was shortest in older compared to other groups (median: 9 months vs. 10 months in the young and 11.5 months in the middle-aged group).

### 3.8. AEs (Adverse Events)

**Figure 2:**
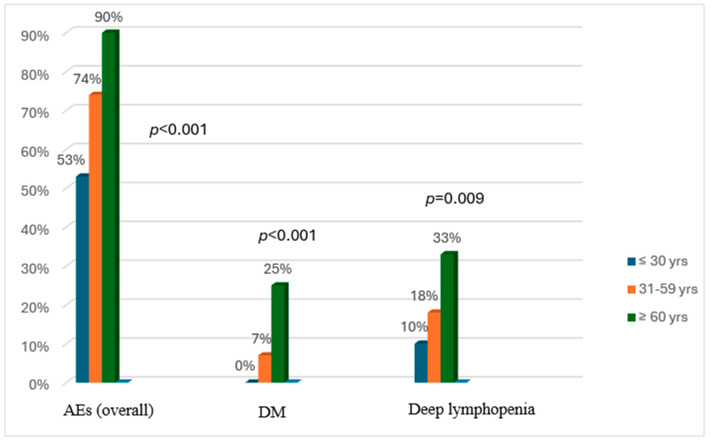
Significant differences in the incidence of overall and selected adverse events (treatment-related diabetes mellitus and deep lymphopenia < 0.5 × 10⁹/L) across age groups. AEs, adverse events; DM, diabetes mellitus.

Adverse events (AEs) after treatment were observed in 72% of the patients (189/264), and their frequency significantly increased with the age of GPA onset (53% vs. 73% vs. 90%; *p* < 0.001). The most common AEs were infections (mainly respiratory) (26%) and lymphopenia < 1 × 10^9^/L (25%), followed by deep lymphopenia < 0.5 × 10^9^/L (19%), leukopenia (17%), severe infections (12%), deep vein thrombosis (DVT) (9%), DM (8%), deaths (4%), hemorrhagic cystitis (3%), and gastrointestinal bleeding (1%). Deep lymphopenia (<0.5 × 10^9^/L) and DM were identified as AEs significantly more common in the older group than in the young group (33% vs. 10% and 25% vs. 0%; *p* = 0.003 and *p* < 0.001, respectively; Figure 2). DM was also significantly more frequent in the older group than in the middle-aged group (25% vs. 6%; *p* < 0.001). The median early VDI score was 2 (IQR 1.0–3.0), with no significant differences between groups (*p* = 0.131).

## 4. Discussion

Our analysis of a large cohort of patients with GPA revealed that age at diagnosis was associated with specific clinical characteristics. We found that older patients (≥60 years) were more likely to experience peripheral neuropathy compared to younger patients and liver involvement than the middle-aged group. They also had more severe lung diseases and more frequently required biopsies. In turn, patients diagnosed between 31 and 59 years old exhibited the highest disease activity and inflammatory markers. This group was more likely to experience severe anemia and peripheral neuropathy than younger patients, while their lung disease was less severe than that of older patients but more severe than that of younger patients. In contrast, younger patients (≤30 years) generally presented with milder disease, characterized by the least severe lung involvement, mildest anemia, and highest albumin levels. Our findings also indicated that comorbidities in GPA significantly increased with age at diagnosis, with HTN, DM, and cancer being the most common in older patients, while non-cardiovascular comorbidities were more prevalent in the 31–59 age group. Finally, we demonstrated that treatment efficacy and relapse rates did not differ significantly based on age at onset. However, the frequency of AEs significantly increased with age at diagnosis, being highest in older patients.

Only a few studies have compared the clinical characteristics of AAV based on age of onset. However, most of these studies compare “older” patients with “younger” ones, and data specific to GPA are scarce. In these studies, patients diagnosed at an older age had less upper airway involvement [16,23,24,25] and inflammatory arthritis [24], while systemic and neurologic symptoms [24,25] and pulmonary disease [15,16] were more common. Similar to these reports, in our study, neurologic symptoms (manifested as peripheral neuropathy) were also significantly more common in the older and middle-aged groups than in the young-onset patients, and older patients were less likely to experience inflammatory arthritis at baseline. However, contrary to these studies, we found no significant differences in the frequency of pulmonary involvement based on the age at onset, although a significant increase in the severity of lung manifestations with age at GPA onset was observed, with greater lung damage and significantly lower PaO2 at baseline. Additionally, we demonstrated that with older age at disease onset, the need for lung biopsy significantly increases, highlighting diagnostic challenges in older patients, where lung lesions must be differentiated from cancer, requiring invasive diagnostic procedures and more frequent histological confirmation. Furthermore, in our cohort, older patients presented with laryngological symptoms as frequently as younger patients, although hearing loss related to vasculitis at the time of GPA diagnosis was more common in the older group, suggesting a more severe course of these manifestations. Finally, in our study, the highest GPA activity, inflammatory markers, and the most severe anemia (with the lowest hemoglobin concentrations) occurred in the middle-aged group, unlike other reports where these features were more frequently observed in older patients [23,24].

Liver involvement was another manifestation that differed significantly among age groups in our study. Physicians rarely assess liver involvement in GPA because it is not considered a major target organ; therefore, it is often overlooked. The diagnosis of this manifestation requires excluding other causes of hepatotoxicity, as liver biopsy is often unnecessary due to the focal nature of liver injury [26]. A recent report showed frequent liver involvement in AAV patients (49.5%), with the highest prevalence in GPA (55.2%), identified by liver test abnormalities [27], similar to our study. However, in our cohort, liver involvement was observed only in 6% of patients, being significantly more common in older-onset patients (12%) than in the middle-aged group (3%), but not in the young group (7%). In all our patients, the manifestation was mild and transient; however, fatal cases are also described [26]. Our findings show that the increase in liver enzymes at baseline in patients with GPA should prompt us to consider whether this could be a manifestation of GPA. Furthermore, liver test abnormalities should not automatically preclude the use of immunosuppressive drugs due to their potential hepatotoxicity.

In contrast to other studies [16,24], we did not find a significant correlation between the rate of renal involvement and the age at onset in patients with GPA. Median creatinine levels increased with age at GPA diagnosis, with the greatest differences observed between the young and middle-aged groups, but not in the older group. Hemodialysis at baseline was required in only 3% of the patients, primarily in the middle-aged group. Several factors may have influenced these results. First, the number of patients with older-onset disease was relatively small. Additionally, only 5% of patients were ANCA-MPO-positive, while 6% tested positive for pANCA (perinuclear pattern), which was primarily observed in the older group. Higher rates of glomerulonephritis are reported in MPO-AAV patients [28,29], with over 80% experiencing kidney disease at initial presentation, often progressing to chronic kidney injury and adversely affecting survival [6,29]. Finally, the specificity of our cohort, diagnosed and treated in the pulmonology department, may partly explain our results, as patients with severe renal disease may be referred to nephrology centers. However, the frequency of kidney involvement at diagnosis in our study was similar to another large cohort of patients with GPA (57 vs. 56%), though the need for hemodialysis was slightly lower (3% vs. 6%) [30].

Our study showed an increase in comorbidities with age at the onset of GPA, which is consistent with previous reports [24,31]. In our cohort, overall comorbid conditions were present at baseline in approximately 60% of patients, with the highest prevalence observed in the older-onset group, where 88% of these patients had at least one comorbid condition at the time of GPA diagnosis. Preexisting comorbidities, particularly in older patients with common non-specific vasculitis symptoms [12,32], can significantly delay diagnosis [33,34,35]. In our study, the median diagnostic delay was 4 months (range 2–10 months), with the longest delays observed in the older-onset group. However, Monti et al. [24] reported longer diagnostic delays in younger patients compared to older ones. The authors speculated that older patients with preexisting comorbidities may seek medical attention earlier than younger individuals who were previously healthy. Additionally, the study suggested that more severe clinical manifestations in older patients might explain the time to diagnosis [24].

Regarding treatment, we found no significant age-related differences in prescribing practices, treatment duration, or efficacy in GPA patients. However, the oral CYC dosage and initial CS dose were lower in older-onset patients compared to younger ones. The reasons for administering lower doses to older patients, beyond age and renal function [19,20], include comorbidities and the anticipated increased risk of treatment-related side effects, especially infections. Studies comparing drug distribution, doses, and treatment efficacy based on age at GPA onset are limited and inconsistent. In a study by Chen et al. [15], older AAV patients were more likely to receive CS therapy alone for induction, and those who received CYC had a shorter duration of IS therapy and were less likely to respond to it compared to younger patients. In another study, an induction regimen limiting CS exposure and using fixed low-dose CYC pulses in older patients did not affect remission rates or relapse but reduced severe AEs compared to conventional therapy [36]. Conversely, in the study by Thietart et al. [37], patients aged 75 and older had a lower risk of relapse than those aged 65 to 75, despite a lower likelihood of receiving maintenance treatment. In our study, treatment response, remission duration, and relapse rates were similar across age groups, aligning with the findings of several other studies on this topic [14,15,36,38].

Finally, we showed that the frequency of AEs increases significantly with age at GPA onset. Our results are consistent with most other reports [14,15,32]. Overall, in our cohort, AEs affected 72% of the patients during a median follow-up of 12.5 years. For comparison, in another analysis of a cohort of patients with mixed AAV, AEs occurred in 60% of the patients during a shorter observation period (4.0 years) [39]. In agreement with other reports [14,15,39,40,41], the most common AEs in our study were infections, identified in 26% of all patients, with severe infections occurring in 12%. However, in contrast to these studies, the frequency of infection rates in our cohort was comparable between age groups and was not significantly more common in older patients [14,15,41,42]. Interestingly, treatment-related deep lymphopenia (<0.5 × 10^9^/L) in our cohort was significantly more common in older patients than in younger patients; however, this did not result in a higher infection rate in this group. Similar observations were reported by Berti et al. [14] who found that older patients with AAV had a higher rate of severe infections than younger patients, but overt leukopenia or lymphopenia was not associated with infection in these patients, suggesting that IS may have further contributed to immunologic abnormalities, predisposing elderly patients to severe infections [14]. Krafcik et al. [16], who presented results similar to ours, compared younger- and older-onset GPA patients, where the overall incidence of infectious treatment complications was also similar between the groups. However, the risk of mortality from infection was substantially higher in elderly patients. Interestingly, Berti et al. [14] demonstrated a significant increase in treatment-related rates of cardiovascular complications in patients with AAV during follow-up (including DM), but without significant differences in their frequency between young and elderly patients. In our study, treatment-related DM was significantly more common in older patients than in younger patients, suggesting that older patients may be more sensitive to this complication of CS exposure. AAV has been identified as a risk factor for cardiovascular disease, with the risk of complications increasing over time [36,43]. Moreover, recent data indicate an association between AAV and an elevated risk of cardiovascular disease in the months leading up to diagnosis, highlighting the importance of early clinical awareness in cardiovascular health [44].

There are several limitations to consider in this study. First, the single-center design may influence the results due to the involvement of a specific group of GPA patients diagnosed at the pneumological department. Another limitation is the retrospective nature of this study, as the retrospective data collection may have led to inconsistencies or missing data. This data shortage primarily concerned laboratory test results; inflammatory markers were unavailable in up to 60% of the patients, and data on immunoglobulin concentrations were missing. Additionally, data regarding the targeting antigen of ANCA (PR3 or MPO) were missing in 63.5% of patients (167/263), largely because of ELISA’s unavailability and limited use. Most of our patients had cANCA, and of those in whom the ANCA antigen was determined, most were PR3 positive, which may have affected the clinical manifestations and treatment outcomes. In addition, the large disproportion of PR3- and MPO-positive patients (88 vs. 5%) made it impossible to compare patients by ANCA stratification. Next, the groups of patients were not equal numerically, especially the middle-aged group, which was the largest compared to the other groups, which may have prevented some differences from being revealed. Finally, most patients were treated with CYC, limiting our results’ generalizability to patients with induced rituximab.

However, this study also has several strengths. Given the monocentric design and rarity of the disease, this study included a large number of patients with GPA with specific characteristics such as common lung involvement (92%), relatively rare renal impairment (25%), and very rare limited disease (6%). A strength of this study is that the diagnosis of GPA was established and confirmed in every case at a single referral center, minimizing the likelihood of overdiagnosis. Similarly, as patient care was provided in one center, treatment strategies and management were largely standardized. Another strength is the prolonged observation period (median 12.5 years), which allowed for monitoring treatment results and relapses in long-term follow-up. Finally, although the specific phenotype of our patients can be considered a limitation, our study demonstrated variations in clinical presentations and treatment outcomes based on the age at disease onset in a well-defined GPA cohort, mainly with PR3-ANCA (and/or cANCA) positivity, recruited at the pneumological department.

## 5. Conclusions

In conclusion, our study highlighted age-specific characteristics in a well-defined, monocentric cohort of patients with newly diagnosed GPA. Understanding these age-related differences in clinical presentations could facilitate more efficient diagnoses and the development of tailored therapeutic strategies aimed at optimizing treatment outcomes and minimizing complications. Further studies involving a larger and more diverse cohort of GPA patients are needed to confirm these findings.

## Figures and Tables

**Table 1:** Clinical characteristics of study patients with pairwise comparisons across age groups.

Parameter	All		≤30 yrs I Group		31–59 yrs II Group		≥60 yrs III Group		*p*-Value ^A^	*p*-Value ^B^ Pairwise Comparison Across Age Groups
	n	n (%)	n	n (%)	n	n (%)	n	n (%)		
1. Sex (female)	264	137 (52%)	59	33 (56%)	156	74 (47%)	49	30 (61%)	0.189	-
2. Upper airway	264	221 (84%)	59	52 (88%)	156	132 (85%)	49	37 (77%)	0.267	-
Nose	264	206 (78%)	59	49 (83%)	156	121 (78%)	49	36 (73%)	0.477	-
- crusting	264	106 (40%)	59	19 (32%)	156	65 (42%)	49	22 (45%)	0.340	-
- epistaxis	264	71 (27%)	59	20 (34%)	156	37 (24%)	49	14 (29%)	0.310	-
- cartilage destruction/sadle nose	264	21 (8%)	59	8 (14%)	156	11 (7%)	49	2 (4%)	0.157	-
- other (discharge, obstruction)	264	74 (28%)	59	16 (27%)	156	47 (30%)	49	11 (22%)	0.571	-
histology	264	78 (30%)	59	19 (32%)	156	45 (29%)	49	14 (29%)	0.878	-
Sinuses	264	143 (54%)	59	32 (54%)	156	86 (55%)	49	25 (51%)	0.881	-
Ears	264	95 (36%)	59	23 (39%)	156	56 (36%)	49	26 (33%)	0.832	-
- hearing loss at time of GPA diagnosis	264	18 (7%)	59	1 (2%)	156	10 (6%)	49	7 (14%)	0.034 **	0.292 (I–II) 0.034 (I–III) * 0.148 (II–III)
3. Lower airway	264	113 (43%)	59	27 (46%)	156	65 (42%)	49	21 (43%)	0.760	-
Bronchi	264	91 (34%)	59	19 (32%)	156	55 (35%)	49	17 (35%)	0.971	-
- inflammation/stenosis	264	65 (25%)	59	16 (27%)	156	36 (23%)	49	13 (27%)	0.705	-
- ulceration/necrosis	264	27 (10%)	59	4 (7%)	156	17 (11%)	49	6 (13%)	0.629	-
- other (mucosal anbormalities, polyps)	264	22 (8%)	59	1 (2%)	156	18 (12%)	49	3 (6%)	0.062	-
histology	264	47 (18%)	59	10 (17%)	156	27 (17%)	49	10 (20%)	0.872	-
Trachea	264	44 (17%)	59	11 (19%)	156	26 (17%)	49	7 (14%)	0.765	-
- SGS	264	39 (15%)	59	10 (17%)	156	22 (14%)	49	7 (14%)	0.803	-
- the need of IDIT	264	12 (5%)	59	4 (7%)	156	7 (5%)	49	1 (2%)	0.474	-
- other (mucosal abnormalities, ulcerations)	264	9 (3%)	59	2 (3%)	156	7 (5%)	49	0 (0%)	0.318	-
histology	264	17 (6%)	59	5 (9%)	156	10 (6%)	49	2 (4%)	0.605	-
4. Lungs	264	242 (92%)	59	55 (93%)	156	144 (92%)	49	43 (88%)	0.535	-
- masses/nodules/cavities	264	162 (61%)	59	35 (59%)	156	95 (61%)	49	32 (65%)	0.803	-
- infiltrates/consolidations	264	105 (40%)	59	28 (47%)	156	63 (40%)	49	14 (29%)	0.132	-
- DAH	264	49 (19%)	59	11 (19%)	156	33 (21%)	49	5 (10%)	0.228	-
- other (e.g., ground-glass opacities)	264	22 (8%)	59	6 (10%)	156	33 (21%)	49	7 (14%)	0.144	-
the need of invasive ventilation support	264	12 (5%)	59	4 (7%)	156	6 (4%)	49	2 (4%)	0.644	-
unilateral lung lesions	264	14 (5%)	59	6 (10%)	156	5 (3%)	49	3 (6%)	0.125	-
isolated lung lesions	264	7 (3%)	59	0 (0%)	156	3 (2%)	49	4 (8%)	0.021 **	0.673 (I–II) 0.084 (I–III) 0.100 (II–III)
histology	264	59 (22%)	59	6 (10%)	156	38 (24%)	49	15 (31%)	0.026 **	0.021 (I–II) * 0.008 (I–III) ** 0.383 (II–III)
5. Pleura	264	9 (3%)	59	3 (5%)	156	6 (4%)	49	0 (0%)	0.313	-
6. Lymph nodes	264	15 (5%)	59	5 (9%)	156	6 (4%)	49	4 (8%)		-
7. Oral cavity	264	46 (17%)	59	11 (19%)	156	29 (19%)	49	6 (12%)	0.571	-
- erosions/ulcers	264	38 (14%)	59	10 (17%)	156	23 (15%)	49	5 (10%)	0.599	-
- petechiae	264	10 (4%)	59	3 (5%)	156	6 (4%)	49	1 (2%)	0.710	-
- gingival hiperplasia	264	4 (2%)	59	2 (3%)	156	2 (1%)	49	0 (0%)	0.333	-
histology	264	4 (2%)	59	0 (0%)	156	4 (3%)	49	0 (0%)	0.245	-
8. Kidney	264	150 (57%)	59	35 (59%)	156	92 (59%)	49	23 (47%)	0.302	-
- hematuria	264	138 (52%)	59	33 (56%)	156	83 (53%)	49	22 (45%)	0.487	-
- proteinuria	264	118 (45%)	59	24 (41%)	156	76 (49%)	49	18 (38%)	0.264	-
- renal insufficiency	264	66 (25%)	59	9 (15%)	156	44 (28%)	49	13 (27%)	0.142	-
- the need of HD	264	7 (3%)	59	0 (0%)	156	6 (4%)	49	1 92%)	0.281	-
histology	264	9 (3%)	59	3 (5%)	156	6 (4%)	49	0 (0%)	0.313	-
9. Eye	264	63 (24%)	59	13 (22%)	156	41 (26%)	49	9 (18%)	0.556	-
- conjunctivitis	264	45 (17%)	59	9 (15%)	156	31 (20%)	49	5 (10%)	0.307	-
- episcleritis	264	13 (5%)	59	4 (8%)	156	7 (4%)	49	2 (4%)	0.764	-
- scleritis	264	5 (2%)	59	0 (0%)	156	4 (3%)	49	1 (2%)	0.468	-
- uveitis/retinitis	264	9 (3%)	59	3 (5%)	156	3 (2%)	49	3 (6%)	0.248	-
- lacrimal gland	264	7 (3%)	59	1 (2%)	156	4 (3%)	49	2 (4%)	0.713	-
- orbital mass	264	9 (3%)	59	2 (3%)	156	5 (3%)	49	2 (4%)	0.942	-
histology	264	0 (0%)	59	0 (0%)	156	0 (0%)	49	0 (0%)	1	-
10. Skin	264	76 (29%)	59	20 (34%)	156	48 (31%)	49	8 (16%)	0.092	-
- purpura	264	59 (22%)	59	14 (24%)	156	38 (24%)	49	7 (14%)	0.322	-
- ulcerations	264	18 (7%)	59	3 (5%)	156	14 (9%)	49	1 (2%)	0.204	-
- nodules	264	10 (43%)	59	3 (5%)	156	7 (4%)	49	0 (0%)	0.300	-
- necrosis	264	12 (5%)	59	3 (5%)	156	8 (5%)	49	1 (2%)	0.661	-
- other (e.g.,urticaria, petechiae)	264	12 (5%)	59	4 (7%)	156	6 (4%)	49	2 (4%)	0.648	-
histology	264	16 (6%)	59	3 (5%)	156	10 (6%)	49	3 (6%)	0.936	-
11. Nervous system	264	41 (16%)	59	8 (14%)	156	23 (15%)	49	10 (20%)	0.566	-
- meningitis	264	6 (2%)	59	3 (5%)	156	3 (2%)	49	0 (0%)	0.190	-
- bleeding	264	1 (0%)	59	0 (0%)	156	1 (1%)	49	0 (0%)	0.706	-
- vasculitic lesions	264	8 (3%)	59	0 (0%)	156	6 (4%)	49	2 (4%)	0.304	-
- disturbances of consciousness	264	2 (1%)	59	0 (0%)	156	2 (1%)	49	0 (0%)	0.498	-
- convulsions	264	2 (1%)	59	0 (0%)	156	2 (1%)	49	0 (0%)	0.498	-
- cranial nerves	264	6 (2%)	59	0 (0%)	156	6 (4%)	49	0 (0%)	0.119	-
- peripheral neuropathy	264	20 (8%)	59	0 (0%)	156	15 (10%)	49	5 (10%)	0.044 **	0.014 (I–II) ** 0.012 (I–III) ** 0.904 (II–III)
histology	264	0 (0%)	59	0 (0%)	156	0 (0%)	49	0 (0%)	1	-
12. Digestive tract	263	30 (11%)	59	8 (14%)	156	15 (10%)	49	7 (14%)	0.568	-
- bleeding	263	9 (3%)	59	2 (3%)	156	7 (4%)	49	0 (0%)	0.320	-
- stomach pain	263	5 (2%)	59	3 (5%)	156	2 (1%)	49	0 (0%)	0.106	-
- liver	263	15 (6%)	59	4 (7%)	156	5 (3%)	49	6 (12%)	0.053 *	0.243 (I–II) 0.329 (I–III) 0.014 (II–III) **
- other (e.g., splenomegaly)	263	3 (1%)	59	2 (3%)	156	1 (1%)	49	0 (0%)	0.168	-
histology	263	3 (1%)	59	0 (0%)	156	2 (1%)	49	1 (2%)	0.587	-
13. Heart and/or pericardium	264	16 (6%)	59	2 (4%)	156	10 (6%)	49	4 (8%)	0.562	-
- myocarditis	264	8 (3%)	59	1 (2%)	156	5 (3%)	49	2 (4%)	0.756	-
- ischemia/infarction	264	5 (2%)	59	0 (0%)	156	5 (3%)	49	0 (0%)	0.171	-
- pericarditis	264	7 (3%)	59	2 (3%)	156	2 (1%)	49	3 (6%)	0.170	-
14. Arthritis	264	51 (19%)	59	15 (25%)	156	41 (27%)	49	5 (10%)	0.055 *	0.859 (I–II) 0.043 (I–III) * 0.017 (II–III) *
15. General symptoms	264	220 (83%)	59	52 (88%)	156	129 (83%)	49	39 (80%)	0.468	-
- weight loss	264	133 (50%)	59	26 (44%)	156	83 (53%)	49	24 (49%)	0.478	-
- fever	264	159 (60%)	59	37 (63%)	156	97 (62%)	49	25 (51%)	0.344	-
- arthralgia	264	62 (23%)	59	17 (29%)	156	38 (24%)	49	7 (14%)	0.191	-
- myalgia	264	9 (3%)	59	2 (3%)	156	7 (4%)	49	0 (0%)	0.320	-
- fatigue	264	24 (9%)	59	5 (8%)	156	14 (9%)	49	5 (10%)	0.950	-
16. Other rare symptoms (pituitary gland, large vessels, thyroid, salivary glands, adrenal glands)	264	34 (13%)	59	10 (17%)	156	18 (12%)	49	6 (12%)	0.566	-
17. More than three organs involved	264	147 (56%)	59	36 (61%)	156	89 (57%)	49	22 (45%)	0.211	-
18. Limited disease	264	17 (6%)	59	3 (5%)	156	9 (6%)	49	5 (10%)	0.452	-
19. ANCA positive	262	232 (88%)	59	52 (88%)	154	139 (90%)	49	41 (84%)	0.449	-
- cANCA	262	185 (71%)	59	45 (76%)	154	108 (70%)	49	32 (65%)	0.451	-
- pANCA	262	16 (6%)	59	1 (2%)	154	8 (5%)	49	7 (14%)	0.019 **	0.253 (I–II) 0.013 (I–III) ** 0.035 (II–III) *
- anti-PR3	96	84 (88%)	29	27 (93%)	46	39 (85%)	21	18 (86%)	0.548	-
- anti-MPO	96	5 (5%)	29	0 (0)%)	46	3 (7%)	21	2 (10%)	0.280	-
20. *S. aureus* nasal carriage	234	100 (43%)	55	26 (47%)	133	61 (46%)	46	13 (28%)	0.085 *	0.860 (I–II) 0.051 (I–III) * 0.037 (II–III) *
21. BVAS/WG (median, IQR)	263	8 (5–12)	59	7 (4–12)	155	8 (4–14)	49	7 (3–10)	0.020 **	0.281 (I–II) 1 (I–III) 0.029 (II–III) *
22. Delay in diagnosis (mo; median, IQR)	261	4 (2–10)	59	4 (2–10)	153	4 (2–9)	49	6 (3–12)	0.020 **	1 (I–II) 0.025 (I–III) * 0.048 (II–III) *
23. Time of follow-up (yrs; median, IQR)	263	12.5 (8.6–17.8)	59	14.7 (9.8–20.0)	155	12.8 (8.6–17.7)	49	10.0 (6.8–13.7)	0.001 **	0.194 (I–II) 0.001 (I–III) ** 0.021 (II–III) *

^A^*p*-value of <0.05 was considered statistically significant; ^B^ Bonferroni correction, *p*-value of <0.017 was considered statistically significant; double asterisks (**) indicate results statistically significant; a single asterisk (*) indicates results approaching statistical significance. Abbreviations: GPA, granulomatosis with polyangiitis; SGS, subglottic stenosis; IDIT, intratracheal dilation-injection technique; DAH, diffuse alveolar hemorrhage; HD, hemodialysis; ANCA, anti-neutrophil cytoplasmic antibodies; cANCA, cytoplasmic labeling pattern of ANCA; pANCA, perinuclear labeling pattern of ANCA; anti-PR3, anti-proteinase 3; anti-MPO, anti-myeloperoxidase; BVAS/WG, Birmingham Vasculitis Activity Score for Wegener’s Granulomatosis; IQR, interquartile range.

**Table 2:** Comparison of laboratory results across age groups.

Parameter		All	≤30 yrs		31–59 yrs		≥60 yrs		*p*-Value ^A^	*p*-Value ^B^ Pairwise Comparison Across Age Groups
	n	Median (IQR)	n	Median (IQR) I Group	n	Median (IQR) II Group	n	Median (IQR) III Group		
Leukocytes (×10^9^/L)	260	11.5 (8.3–15.1)	57	11.9 (8.7–16.0)	154	11.7 (8.5–15.0)	49	10.3 (7.3–13.9)	0.202	-
Lymphcytes (×10^9^/L)	251	1.7 (1.3–2.2)	56	1.6 (1.2–2.3)	147	1.8 (1.4–2.4)	48	1.5 (1.1–1.9)	0.016 **	0.861 (I–II) 0.353 (I–III) 0.013 (II–III) **
Hemoglobin (g/dL)	260	10.5 (8.6–12.0)	57	11.3 (10.2–12.9)	154	9.9 (8.4–11.5)	49	10.7 (8.7–12.1)	0.003 **	0.003 (I–II) ** 0.363 (I–III) 0.570 (II–III)
Platelet (×10^9^/L)	249	410 (300–557)	57	410 (293–530)	144	427 (315–600)	48	380 (260–444)	0.075	-
Total protein (g/dL)	234	7.0 (6.5–7.5)	54	7.1 (6.7–7.8)	137	7.0 (6.4–7.4)	43	6.9 (6.4–7.5)	0.070	-
Albumin (g/dL)	209	3.1 (2.7–3.6)	49	3.5 (3.0–4.0)	121	2.9 (2.6–3.5)	39	3.0 (2.6–3.5)	0.001 **	0.001 (I–II) ** 0.016 (I–III) ** 1 (II–III)
Globulin (g/dL)	192	1.3 (1.0–1.6)	43	1.4 (1.0–1.6)	117	1.3 (1.0–1.6)	32	1.2 (1.0–1.7)	0.964	-
Fibrinogen (g/dL)	182	6.2 (2.0–2.2)	40	5.2 (3.7–7.4)	104	6.4 (5.1–7.6)	38	6.5 (4.9–7.4)	0.146	-
Creatinine (mg/dL)	259	0.9 (0.8–1.3)	57	0.8 (0.7–1.0)	154	0.9 (0.8–1.4)	48	1.0 (0.8–1.4)	0.029 **	0.039 (I–II) * 0.083 (I–III) 1 (II–III)
Pa0_2_ (mmHg)	183	73.0 (65.0–79.9)	41	79.0 (72.2–88.4)	110	72.0 (65.0–79.0)	32	69.0 (62.5–74.5)	<0.001 **	0.001 (I–II) ** <0.001 (I–III) ** 0.299 (II–III)
CRP (mg/L)	102	46.2 (11.1–127.0)	24	18.6 (5.0–88.6)	56	79.6 (19.3–145.5)	22	34.0 (14.0–117.9)	0.052 *	0.046 (I–II) * 0.632 (I–III) 1 (II–III)

^A^*p*-value of <0.05 was considered statistically significant; ^B^ Bonferroni correction, *p*-value of <0.017 was considered statistically significant; double asterisks (**) indicate results statistically significant; a single asterisk (*) indicates results approaching statistical significance. Abbreviations: Pa02, partial pressure of oxygen; CRP, C-reactive protein; IQR, interquartile range.

**Table 3:** Prevalence and distribution of baseline comorbid conditions according to age groups.

Parameter	All		≤30 yrs I Group		31–59 yrs II Group		≥60 yrs III Group		*p*-Value ^A^	*p*-Value ^B^ Pairwise Comparison Across Age Groups
	n	n (%)	n	n (%)	n	n (%)	n	n (%)		
Comorbidities (overall)	264	153 (58%)	59	21 (36%)	156	90 (58%)	49	42 (88%)	<0.001 **	0.003 (I–II) ** <0.001 (I–III) ** <0.001 (II–III) **
- HTN	264	52 (20%)	59	7 (12%)	156	25 (16%)	49	20 (41%)	<0.001 **	0.444 (I–II) <0.001 (I–III) ** <0.001 (II–III) **
- DM	264	14 (5%)	59	1 (2%)	156	5 (3%)	49	8 (16%)	<0.001 **	0.549 (I–II) **0.017 (I**–III) * 0.003 (II–III) **
- IHD	264	10 (4%)	59	0 (0%)	156	6 (4%)	49	4 (8%)	0.086	-
- COPD	264	4 (2%)	59	0 (0%)	156	2 (1%)	49	2 (4%)	0.209	-
- cancer	264	5 (2%)	59	0 (0%)	156	1 (1%)	49	4 (8%)	0.002 **	0.538 (I–II) 0.085 (I–III) 0.014 (II–III) **
- DVT	264	9 (3%)	59	4 (7%)	156	4 (3%)	49	1 (2%)	0.265	-
- depression	264	8 (3%)	59	0 (0%)	156	3 (2%)	49	5 (10%)	0.004 **	0.283 (I–II) **0.040 (I**–III) * 0.029 (II–III) *
- other	264	70 (27%)	59	10 (17%)	156	50 (32%)	49	10 (20%)	0.046 **	0.028 (I–II) * 0.645 (I–III) 0.118 (II–III)

^A^*p*-value of <0.05 was considered statistically significant; ^B^ Bonferroni correction, *p*-value of <0.017 was considered statistically significant; double asterisks (**) indicate results statistically significant; a single asterisk (*) indicates results approaching statistical significance. Abbreviations: HTN, hypertension; DM, diabetes mellitus; IHD, ischemic heart disease; DVT, deep venous thrombosis.

**Table 4:** Comparison of treatment strategies and outcomes across age groups.

Parameter	All		≤30 yrs I Group		31–59 yrs II Group		≥60 yrs III Group		*p*-Value ^A^	*p*-Value ^B^ Pairwise Comparison Across Age Groups
	n	n (%)	n	n (%)	n	n (%)	n	n (%)		
1. Induction	264		59		156		49			
- CYC orally		210 (80%)		46 (79%)		120 (77%)		44 (90%)	0.153	-
- CYC infusions		19 (7%)		3 (5%)		16 (10%)		0 (0%)		
- other IS		10 (4%)		1 (2%)		9 (6%)		0 (0%)		
- only CS		25 (10%)		9 (15%)		11 (7%)		5 (10%)		
- CS intravenously		105 (40%)		21 (36%)		63 (40%)		21 (43%)		
2. Maintenance	264		59		156		49		0.518	-
- CYC		105 (40%)		23 (39%)		65 (42%)		17 (35%)		
- MTX		31 (12%)		8 (13.5%)		21 (13%)		2 (4%)		
- AZA		96 (36%)		19 (32%)		55 (35%)		22 (45%)		
- only CS		32 (12%)		9 (15%)		15 (9%)		8 (16%)		
3. Dosage and duration	n	median (IQR)	n	median (IQR)	n	median (IQR)	n	median (IQR)		
- oral dosage of CYC (mg/kg/day)	210	2.0 (2.0–2.2)	46	2.0 (2.0–2.2)	120	2.0 (2.0–2.2)	44	2.0 (1.8–2.0)	0.023 **	1 (I–II) 0.047 (I–III) * 0.072 (II–III)
- duration of treatment with CYC (mo)	232	8.8 (5.5–15.8)	44	9.5 (6.0–16.0)	128	8.0 (5.5–16.0)	45	8.0 (5–14)	0.579	-
- cumulative dose of CYC (g)	233	28 (15–47)	48	31 (18–46)	139	27 (15–50)	45	26 (14–42)	0.826	-
- starting dose with CS (mg/kg/day of prednisolone equivalent)	251	1.0 (0.7–1.0)	50	1.0 (0.8–1.0)	138	1.0 (0.7–1.0)	49	0.8 (0.6–1.0)	0.015 **	0.612 (I–II) 0.027 (I–III) * 0.185 (II–III)
- dose >15 mg/day (mo)	236	7 (5–11)	58	8 (5–12)	144	7 (5–11)	46	7 (6–10)	0.818	-
- total duration of CS use (mo)	249	19 (12–24)	56	18 (11–24)	134	19 (12–24)	48	19 (12–24)	0.855	-
- total duration of IS (mo)	262	17 (11–24)	57	18 (11–24)	153	16 (11–23)	49	19 (12–24)	0.463	-
4. Other treatment	n	n (%)	n	n (%)	n	n (%)	n	n (%)		
- T/S	264	57 (22%)	59	22 (37%)	156	27 (17%)	49	8 (16%)	0.004 **	0.002 (I–II) ** 0.015 (I–III) ** 0.873 (II–III)
- IVIG	264	14 (5%)	59	6 (10%)	156	7 (4%)	49	1 (2%)	0.140	-
- plasmapheresis	264	8 (3%)	59	3 (5%)	156	4 (3%)	49	1 (2%)	0.569	-
- HD	264	5 (2%)	59	0 (0%)	156	5 (3%)	49	0 (0%)	0.171	-
- blood transfusion	264	30 (11%)	59	4 (7%)	156	24 (15%)	49	2 (4%)	0.043 **	0.094 (I–II) 0.542 (I–III) 0.038 (II–III) *
- chloramphenicol	264	3 (1%)	59	0 (0%)	156	2 (1%)	49	1 (2%)	0.587	-
- kidney transplantation	264	1 (0%)	59	0 (0%)	156	1 (1%)	49	0 (0%)	0.706	-
5. Treatment efficacy	264		59		156		49			-
- remission		206 (78%)		45 (76%)		121 (78%)		39 (80%)	0.564	-
- minimally persistent activity of the disease		42 (16%)		11 (19%)		24 (15%)		6 (12%)		
-failure		12 (5%)		3 (5%)		5 (3%)		4 (8%)		
- unknown		3 (1%)		0 (0%)		3 (2%)		0 (0%)		
Negatization of ANCA	255	164 (64%)	58	39 (67%)	151	93 (62%)	46	32 (70%)	0.533	-
VDI (median; IQR)	264	2.0 (1.0–3.0)	59	1.0 (0.0–2.0)	156	2.0 (1.0–3.0)	49	2.0 (1.0–3.0)	0.131	-
Relapses (YES), No of pts (%)	264	153 (58%)	59	34 (58%)	156	94 (60%)	49	25 (51%)	0.520	
Number of relapses (median; IQR)	264	1 (0–2)	59	1 (0–3)	156	1 (0–2)	49	1 (0–2)	0.269	-
Time to first relapse (mo; median; IQR)	153	11 (4–26)	34	10 (4–20)	94	11.5 (3–35)	49	9 (4–22)	0.657	-

^A^*p*-value of <0.05 was considered statistically significant; ^B^ Bonferroni correction, *p*-value of <0.017 was considered statistically significant; double asterisks (**) indicate results statistically significant; a single asterisk (*) indicates results approaching statistical significance. Abbreviations: CYC, cyclophosphamide; IS, immunosuppression; CS, corticosteroids; MTX, methotrexate; AZA, azathioprine; T/S, trimethoprim/sulfamethoxazole; IVIG, intravenous immune globulin; HD, hemodialysis; ANCA, anti-neutrophil cytoplasmic antibodies; VDI, vasculitis damage index; IQR, interquartile range.

## Data Availability

The data presented in this study are available upon request from the corresponding author. These data are not publicly available because of privacy restrictions.

## Abbreviations

The following abbreviations are used in this manuscript:

GPA	Granulomatosis with Polyangiitis
ANCA	Anti-neutrophil Cytoplasmic Antibody
PR3	Proteinase 3
MPO	Myeloperoxidase
MPA	Microscopic Polyangiitis
EGPA	Eosinophilic Granulomatosis with Polyangiitis
AAV	ANCA-Associated Vasculitis
BVAS/WG	Birmingham Vasculitis Activity Score for Wegener’s Granulomatosis
IS	Immunosuppression/Immunosuppressive
CS	Corticosteroids
CYC	Cyclophosphamide
SGS	Subglottic stenosis
IV	Intravenous
HTN	Hypertension
DM	Diabetes Mellitus
cANCA	Cytoplasmic labeling pattern of Anti-neutrophil Cytoplasmic Antibodies
pANCA	Perinuclear labeling pattern of Anti-neutrophil Cytoplasmic Antibodies
T/S	Trimethoprim/Sulfamethoxazole
*S. aureus*	*Staphylococcus aureus*
STATISTICA PL	Statistical Software by StatSoft Poland, version 13
IQR	Interquartile Range

## References

[1] Jennette J.C., Falk R.J., Andrassy K., Bacon P.A., Churg J., Gross W.L., Hagen E.C., Hoffman G.S., Hunder G.G., Kallenberg C.G. (1994). Nomenclature of systemic vasculitides. Proposal of an international consensus conference. Arthritis Rheum.

[2] Jennette J.C., Falk R.J., Bacon P.A., Basu N., Cid M.C., Ferrario F., Flores-Suarez L.F., Gross W.L., Guillevin L., Hagen E.C. (2013). 2012 revised International Chapel Hill Consensus Conference Nomenclature of Vasculitides. Arthritis Rheum.

[3] Lynch J.P., Derhovanessian A., Tazelaar H., Belperio J.A. (2018). Granulomatosis with polyangiitis (Wegener’s granulomatosis): Evolving concept in treatment. Semin. Respir. Crit. Care Med..

[4] Kronbichler A., Bajema I.M., Bruchfeld A., Mastroianni Kirsztajn G., Stone J.H. (2024). Diagnosis and management of ANCA-associated vasculitis. Lancet.

[5] Miloslavsky E.M., Lu N., Unizony S., Choi H.K., Merkel P.A., Seo P., Spiera R., Langford C.A., Hoffman G.S., Kallenberg C.G.M. (2016). Myeloperoxidase-antineutrophil cytoplasmic antibody (ANCA)-positive and ANCA-negative patients with granulomatosis with polyangiitis (Wegener’s). Distinct patients subsets. Arthritis Rheumatol..

[6] Alba M.A., Jennette J.C., Hu Y., Poulton C.J., Blazek L., Derebail V.K., Falk R.J., Hogan S.L. (2022). Relevance in combined clinicopathologic phenotype and antineutrophil cytoplasmic autoantibody serotype in the diagnosis of antineutrophil cytoplasmic autoantibody vasculitis. Kidney Int. Rep..

[7] Lyons P.A., Rayner T.F., Trivedi S., Holle J.U., Watts R.A., Jayne D.R.W., Baslund B., Brenchley P., Bruchfeld A., Chaudhry A.N. (2012). Genetically distinct subsets within ANCA-associated vasculitis. N. Engl. J. Med..

[8] Watts R.A., Hatemi G., Burns J.C., Mohammad A.J. (2022). Global epidemiology of vasculitis. Nat. Rev. Rheumatol..

[9] Bataille P.M., Durel C.-A., Chauveau D., Panes A., Thervet É.S., Terrier B. (2022). Epidemiology of granulomatosis with polyangiitis and microscopic polyangiitis in adults in France. J. Autoimmun..

[10] Rathmann J., Segelmark M., Englund M., Mohammad A.J. (2023). Stable incidence but increase in prevalence of ANCA-associated vasculitis in southern Sweden: A 23-year study. RMD Open.

[11] Pearce F.A., Grainge M.J., Lanyon P.C., Watts R.A., Hubbard R.B. (2017). The incidence, prevalence and mortality of granulomatosis with polyangiitis in the UK Clinical Practice Research Datalink. Rheumatology.

[12] Berti A., Caporali R., Montecucco C., Paolazzi G., Monti S. (2019). Aging in primary systemic vasculitis: Implications for diagnosis, clinical manifestations, and management. Drugs Aging.

[13] Potentas-Policewicz M., Fijolek J. (2024). Granulomatosis with polyangiitis: Clinical characteristics and updates in diagnosing. Front. Med..

[14] Berti A., Felicetti M., Monti S., Ortolan A., Padoan R., Brunori G., Bortolotti R., Caporali R., Montecucco C., Schiavon F. (2020). Disease and treatment-related morbidity in young and elderly patients with granulomatosis with polyangiitis and microscopic polyangiitis. Semin. Arthritis Rheum..

[15] Chen M., Yu F., Zhang Y., Zhao M.-H. (2008). Antineutrophil cytoplasmic autoantibody-associated vasculitis in older patients. Medicine.

[16] Krafcik S.S., Covin R.B., Lynch J.P., Sitrin R.G. (1996). Wegener’s granulomatosis in the elderly. Chest.

[17] Leavit R.Y., Fauci A.S., Bloch D.A., Michel B.A., Hunder G.G., Arend W.P., Calabrese L.H., Fries J.F., Lie J.T., Lightfoot R.W. (1990). The American College of Rheumatology 1990 Criteria for the classification of Wegener’s granulomatosis. Arthritis Rheum.

[18] Stone J.H., Hoffman G.S., Merkel P.A., Min Y.I., Uhlfelder M.L., Hellmann D.B., Specks U., Allen N.B., Davis J.C., Spiera R.F. (2001). A disease-specific activity index for Wegener’s granulomatosis: Modification of the Birmingham Vasculitis Activity Score. International Network for the study of the systemic vasculitides (INSSYS). Arthritis Rheum..

[19] Yates M., Watts R.A., Bajema I.M., Cid M.C., Crestani B., Hauser T., Hellmich B., Holle J.U., Laudien M., Little M.A. (2016). EULAR/ERA-EDTA recommendations for the management of ANCA-associated vasculitis. Ann. Rheum. Dis..

[20] Hellmich B., Sanchez-Alamo B., Schirmer J.H., Berti A., Blockmans D., Cid M.C., Holle J.U., Hollinger N., Karadag O., Kronbichler A. (2024). EULAR recommendations for the management of ANCA-associated vasculitis: 2022 update. Ann. Rheum. Dis..

[21] Terrier B., Darbon R., Durel C.A., Hachulla E., Karras A., Maillard H., Papo T., Puechal X., Pugnet G., Quemeneur T. (2020). French recommendations for the management of systemic necrotizing vasculitides (polyarteritis nodosa and ANCA-associated vasculitides). Orphanet J. Rare Dis..

[22] Fijołek J., Wiatr E., Petroniec V., Augustynowicz-Kopec E., Bednarek M., Gawryluk D., Martusewicz-Boros M.M., Modrzewska K., Radzikowska E., Roszkowski-Sliz K. (2018). The presence of staphylococcal superantigens in nasal swabs and correlation with activity of granulomatosis with polyangiitis in own material. Clin. Exp. Rheumatol..

[23] Hoganson D.D., From A.M., Michet C.J. (2008). ANCA vasculitis in the elderly. J. Clin. Rheumatol..

[24] Monti S., Craven A., Klersy C., Montecucco C., Caporali R., Watts R., Merkel P.A., Luqmani R., DCVAS Collaborators (2021). Association between age at disease onset of anti-neutrophil cytoplasmic antibody-associated vasculitis and clinical presentations and short-term outcomes. Rheumatology.

[25] Bloom J.L., Pickett-Nairn K., Silveira L., Fuhlbrigge R.C., Cuthbertson D., Akuthota P., Corbridge T.C., Khalidi N.A., Koening C.L., Langford C.A. (2023). The association between age at diagnosis and disease characteristics and damage in patients with ANCA-associated vasculitis. Arthritis Rheumatol..

[26] Holl-Ulrich K., Klass M. (2010). Wegener’s granulomatosis with granulomatous liver involvement. Clin. Exp. Rheumatol..

[27] Willeke P., Schlüter B., Limani A., Becker H., Schotte H. (2016). Liver involvement in ANCA-associated vasculitis. Clin. Rheumatol..

[28] Puéchal X., Iudici M., Pagnoux C., Cohen P., Hamidou M., Aouba A., Lifermann F., Ruivard M., Aumaître O., Bonnotte B. (2022). Comparative study of granulomatosis with polyangiitis subsets according to ANCA status: Data from the French Vasculitis Study Group Registry. RMD Open.

[29] Arnold S., Kitching A.R., Witko-Sarsat V., Wiech T., Specks U., Klapa S., Comdühr S., Stähle A., Müller A., Lamprecht P. (2024). Myeloperoxidase-specific antineutrophil cytoplasmic antibody-associated vasculitis. Lancet Rheumatol..

[30] Iudici M., Pagnoux C., Courvoisier D.S., Cohen P., Hamidou M., Aouba A., Lifermann F., Ruivard M., Aumaître O., Bonnotte B. (2021). Granulomatosis with polyangiitis: Study of 795 patients from the French Vasculitis Study Group registry. Semin. Arthritis Rheum..

[31] Sarica S.H., Gallacher P.J., Dhaun N., Sznajd J., Harvie J., McLaren J., McGeoch L., Kumar V., Amft N., Erwig L. (2021). Multimorbidity in antineutrophil cytoplasmic antibody-associated vasculitis: Results from a longitudinal, multicenter data linkage study. Arthritis Rheumatol..

[32] Jefferson J.A. (2015). Treating elderly patients with ANCA-associated vasculitis. Clin. J. Am. Soc. Nephrol..

[33] Fijołek J., Wiatr E., Gawryluk D., Langfort R., Roszkowski-Śliż K. (2019). Difficulties in recognizing granulomatosis with polyangiitis (GPA) in elderly patients undergoing diagnostic thoracotomy twice—A report of two cases. Adv. Respir. Med..

[34] Wojcik K., Biedron G., Wawrzycka-Adamczyk K., Bazan-Socha S., Cmiel A., Zdrojewski Z., Masiak A., Czuszynska Z., Majdan M., Jeleniewicz R. (2021). Subphenotypes of ANCA-associated vasculitis identified by latent class analysis. Clin. Exp. Rheumatol..

[35] Sreih A.G., Cronin K., Shaw D.G., Young K., Burroughs C., Kullman J., Machireddy K., McAlear C.A., Merkel P.A. (2021). Diagnostic delays in vasculitis and factors associated with time to diagnosis. Orphanet J. Rare Dis..

[36] Pagnoux C., Quéméneur T., Ninet J., Diot E., Kyndt X., de Wazières B., Reny J.L., Puéchal X., le Berruyer P.Y., Lidove O. (2015). Treatment of systemic necrotizing vasculitides in patients aged sixty-five years or older. Results of a multicenter, open-label, randomized controlled trial of corticosteroid and cyclophosphamide-based induction therapy. Arthritis Rheumatol..

[37] Thietart S., Beinse G., Smets P., Karras A., Philipponnet C., Augusto J.F., El Karoui K.E., Mesbah R., Titeca-Beauport D., Hamidou M. (2022). Patients of 75 years and over with ANCA-associated vasculitis have a lower relapse risk than younger patients: A multicentre cohort study. J. Intern. Med..

[38] Haris Á., Polner K., Arányi J., Braunitzer H., Kaszás I., Mucsi I. (2014). Clinical outcomes of ANCA-associated vasculitis in elderly patients. Int. Urol. Nephrol..

[39] Biedroń G., Włudarczyk A., Wawrzycka-Adamczyk K., Wójcik K., Sznajd J., Zdrojewski Z., Masiak A., Czuszyńska Z., Majdan M., Jeleniewicz R. (2020). Treatment and its side effects in ANCA-associated vasculitides—Study based on POLVAS registry data. Adv. Med. Sci..

[40] Haris Á., Polner K., Arányi J., Braunitzer H., Kaszás I. (2021). Incidence and clinical predictors of infections in patients treated with severe systemic ANCA-associated vasculitis. Physiol. Int..

[41] Lafarge A., Joseph A., Pagnoux C., Puéchal X., Cohen P., Samson M., Hamidou M., Karras A., Quemeneur T., Ribi C. (2020). Predictive factors of severe infections in patients with systemic necrotizing vasculitides: Data from 733 patients enrolled in five randomized controlled trials of the French Vasculitis Study Group. Rheumatology.

[42] Mohammad A.J., Segelmark M., Smith R., Englund M., Nilsson J.Å., Westman K., Merkel P.A., Jayne D.R.W. (2017). Severe infection in antineutrophil cytoplasmic antibody-associated vasculitis. J. Rheumatol..

[43] Massicotte-Azarniouch D., Petrcich W., Walsh M., Canney M., Hundemer G.L., Milman N., Hladunewich M.A., Fairhead T., Sood M.M. (2022). Association of anti-neutrophil cytoplasmic antibody-associated vasculitis and cardiovascular events: A population-based cohort study. Clin. Kidney J..

[44] Nygaard L., Polcwiartek C., Nelveg-Kristensen K.E., Carlson N., Kristensen S., Torp-Pedersen C., Gregersen J.W., DANVAS Investigators (2024). Increased risk of cardiovascular disease preceding diagnosis of incident ANCA-associated vasculitis: A Danish nationwide study. Rheumatology.

